# No evidence for association between *tau *gene haplotypic variants and susceptibility to Creutzfeldt-Jakob disease

**DOI:** 10.1186/1471-2350-8-77

**Published:** 2007-12-11

**Authors:** Pascual Sánchez-Juan, Matthew T Bishop, Alison Green, Claudia Giannattasio, Alejandro Arias-Vasquez, Anna Poleggi, Richard SG Knight, Cornelia M van Duijn

**Affiliations:** 1 Institute for Formation and Research of the Fundación "Marqués de Valdecilla" (IFIMAV), Santander, Spain; 2 Genetic Epidemiology Unit, Epidemiology & Biostatistics department, Erasmus MC, Rotterdam, The Netherlands; 3 Centro de Investigación Biomédica en Red sobre Enfermedades Neurodegenerativas (CIBERNED); 4 National CJD Surveillance Unit, The University of Edinburgh, EH4 2XU Edinburgh, UK; 5 Istituto Superiore di Sanità, Laboratory of Virology, Viale Regina Elena 299, 00161 Rome, Italy

## Abstract

**Background:**

A polymorphism at codon 129 of the prion protein gene (*PRNP*) is the only well-known genetic risk factor for Creutzfeldt-Jakob disease (CJD). However, there is increasing evidence that other loci outside the *PRNP *open reading frame might play a role in CJD aetiology as well.

**Methods:**

We studied tau protein gene (*MAPT*) haplotypic variations in a population of sporadic and variant CJD patients. We tested 6 *MAPT *haplotype tagging SNPs (htSNPs) in a Dutch population-based sample of sporadic CJD (sCJD) patients and a cognitively normal control group of similar age distribution. We genotyped the same polymorphisms in two other sample groups of sCJD cases from Italy and the UK. In addition, we compared *MAPT *haplotypes between sCJD and variant CJD (vCJD) patients.

**Results:**

Single locus and haplotype analyses did not detect any significant difference between sCJD cases and controls. When we compared *MAPT *haplotypes between sCJD and variant CJD (vCJD) patients, we found that two of them were represented differently (H1f: 8% in sCJD versus 2% in vCJD; H1j:1% in sCJD versus 7% in vCJD). However, these two haplotypes were rare in both groups of patients, and taking the small sample sizes into account, we cannot exclude that the differences are due to chance. None of the p-values remained statistically significant after applying a multiple testing correction.

**Conclusion:**

Our study shows no evidence for an association between *MAPT *gene variations and sCJD, and some weak evidence for an association to vCJD.

## Background

Tau protein plays a key role in the pathogenesis of several neurodegenerative disorders. Neurofibrillary tangles (NFTs) consisting of accumulation of truncated and hyperphosphorylated tau are one of the hallmarks of Alzheimer's disease (AD) [1]. NFTs are also present in other neurodegenerative diseases, including progressive supranuclear palsy (PSP), cortico basal degeneration (CBD), Pick's disease, and argyrophilic grain disease and Parkinson's disease (PD) [1-4]. In patients with sporadic CJD, the tau protein is profusely released to the CSF [5]. This process is most likely related to the rapid neuronal damage. Tau quantification with Enzyme Linked Immunosorbent Assay (ELISA) is a very valuable aid for sCJD diagnosis [6]. In contrast to sCJD, in vCJD there is an increase of phosphorylated-tau forms in CSF [7]. The pathogenic implications of this finding are unknown, but it suggests that tau phosphorylation may differ between sCJD and vCJD.

The tau protein is encoded by *MAPT *gene, located in chromosome 17. Mutations in *MAPT *have been identified in frontotemporal dementia (FTD) and pallidopontonigral degeneration [8-13]. There are two common *MAPT *extended haplotypes in Caucasians, H1 and H2. The H1 haplotype has been linked to several sporadic neurodegenerative disorders like PSP [14-16], CBD [17], FTD [18], Parkinson's [19] and some studies suggest to Alzheimer's disease [20].

A recent study using data from the HapMap project [21] identified 6 htSNPs capturing 95% of *MAPT *genetic variability in Caucasians [22]. The same group showed that H1c, one of the H1 subhaplotypes, was linked to late onset AD [20]. In the present study we examined the association between *MAPT *haplotypic variants and risk of sCJD and vCJD.

## Methods

Cases were derived from population-based surveys of CJD carried out by national CJD registries from 1991 to 2005 in Italy (n = 194 sCJD), the United Kingdom (UK) (n = 48 sCJD and 52 vCJD) and the Netherlands (n = 79 sCJD). All three countries are part of the European CJD surveillance network EuroCJD [23]. Only patients of Caucasian origin who fulfilled the WHO diagnostic criteria for definite or probable CJD were included [24]. Definite CJD diagnosis was based on neuropathological examination. Probable cases required an appropriate clinical profile, supported by characteristic findings on MRI, EEG or CSF 14-3-3-protein detection. Whenever possible, all EEGs and MRIs were reviewed by a member of the surveillance system and scored for the presence or absence of typical or characteristic diagnostic features [25,26]. The CSF 14-3-3 immunoassays were performed using Western-blotting [27]. Healthy controls (n = 309) were participants of the Rotterdam Study, which is a population-based study of 7385 subjects aged 55 years or older [28]. Controls are all cognitively normal and from Caucasian origin. Signed informed consent to participate in genetic research, approved by the Medical Ethics Committee of the Erasmus Medical Center, was obtained from all controls and patients relatives.

**Figure 1:**
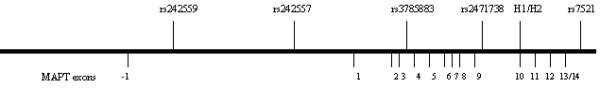
Scheme of *MAPT *genomic structure showing the position of the 6 SNPs genotyped.

For all patients and controls, DNA has been extracted from peripheral leucocytes according to a standard protocol. We genotyped five polymorphisms, which had been previously shown to tag the haplotype diversity of *MAPT *in Caucasians (Figure 1) [22]. Additionally, the H1/H2 clade was defined by typing the SNP g.8117G>A [numbering according to GenBank accession number AC091628.2], with the allele G tagging H1, and the allele A tagging H2 [29]. DNA samples were genotyped with a TaqMan allelic discrimination assay by using the primers shown in Table 1.

**Table 1:** SNP sequence

**SNP**	**Primer sequence**
Rs242559	AGAAAGTTCTCCCAGGAAACAAGAG
	ATTCCTTAGCTGTTACCAGTCACTG

Rs242557	CGTTTCTTCTTCCTTACAAAGCAGTT
	TGTCACGGGACCAGGG

Rs3785883	GCTCAGCGATATTGTCACATGAC
	AGTGTCGGCTGGATGGAC

Rs2471738	AGTGGCCTGGTTAGAGACCTT
	TCTGTCCTGTACCGCAGC

H1/H2	GCCGTCCGCCTCTGT
	CATCGGTCGGGGCCA

Rs7521	CCTGCGTGTCCCATCTACAG
	TCTTCAGCTTTGAAAAGGGTTACCC

**Figure 2:**
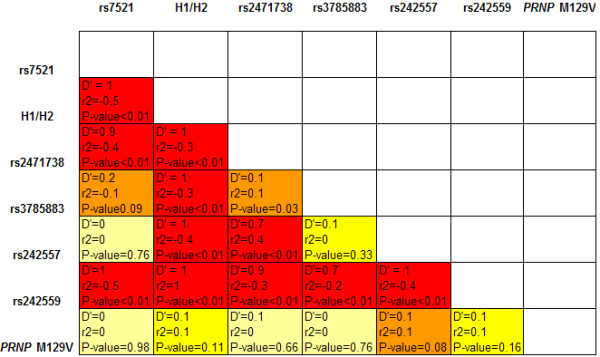
Linkage disequilibrium (LD) plot showing pairwise correlation between SNPs.

Hardy-Weinberg equilibrium (HWE) was calculated for the 6 htSNPs genotypes in the control population using χ^2 ^statistics. We set a significance level of 0.01 in order to take into account the number of tests performed. We assessed pairwise linkage disequilibrium (LD) between the 6 htSNPs using D' and r^2 ^calculated from the expectation-maximization estimated haplotypes (Figure 2). We used the program SNPStats [30] for the LD analysis. Single locus analyses were performed using SPSS software version 13. Allelic and genotypic frequencies were compared using χ^2 ^statistics. Adjusted analyses were performed using multiple logistic regression analysis. Age, gender and *PRNP *M129V genotype were included in the model as covariates. We first compared Dutch sCJD cases and controls using the Italian and UK sCJD populations as replication samples. In a separate analysis we compared UK sCJD with vCJD. Haplotype analyses were performed using the program hplus [31]. We performed haplotype analysis comparing controls versus all sCJD patients and UK sCJD patients with vCJD patients.

## Results

Table 2 shows the basic characteristics of our patients and controls. All htSNPs were in HWE (p > 0.01). Allele frequencies in our control samples were comparable to previous published data [20].

**Table 2:** Descriptive statistics

**Origin**	**Series**	**n**	**% Females**	**% Definite diagnosis**	**Median age (range)**	**PRNP M129V genotype**		
						
						**MM n (%)**	**MV n (%)**	**VV n (%)**
NL	sCJD	79	59	52	67 (34–87)	48 (67)	16 (22)	8 (11)
	controls	309	56	N/A	71 (55–93)	135 (45)	145 (48)	23 (8)

UK	sCJD	48	58	50	67 (49–81)	22 (46)	13 (27)	13 (27)
	vCJD	52	31	74	25 (12–62)	52 (100)	0 (0)	0 (0)

Italy	sCJD	194	57	73	67 (35–87)	141 (73)	31 (16)	22 (11)

In the first analysis we tested whether *MAPT *genetic variations were associated with risk of sCJD. None of the htSNPs genotypes showed a statistically significant association with the disease when comparing the Dutch cases to the matched controls nor when comparing the other non-Dutch patients. Also the pooled analysis was not significant. Adjustment for age, gender and *PRNP *M129V genotype did not alter this conclusion. When we compared in a separate analysis *MAPT *genetic variants between UK sCJD and vCJD, we also failed to find any statistically significant association (Table 3).

**Table 3:** Single locus analysis of the association between *MAPT *and Creutzfeldt-Jakob disease

		**SNP (alleles)**					
		
		rs242559 (A/c)	rs242557 (G/a)	rs3785883 (G/a)	rs2471738 (C/t)	H1/H2 (G/a)	rs7521 (G/a)
**Controls**							
Major allele count (frequences)		467 (0.76)	382 (0.63)	501 (0.82)	495 (0.81)	469 (0.77)	330 (0.54)
	wt	180 (0.60)	123 (0.40)	205 (0.67)	208 (0.68)	183 (0.60)	91 (0.30)
Genotype distribution (frequences)	wt-v	107 (0.29)	136 (0.45)	91 0.30)	79 (0.26)	103 (0.34)	148 (0.48)
	v-v	19 (0.11)	46 (0.15)	10 (0.03)	18 (0.06)	18 (0.06)	67 (0.22)

**NL sCJD**							
Major allele count (frequences)		116 (0.73)	95 (0.60)	134 (0.85)	124 (0.78)	115 (0.73)	88 (0.56)
	wt	47 (0.60)	29 (0.37)	56 (0.71)	50 (0.64)	47 (0.62)	27 (0.35)
Genotype distribution (frequences)	wt-v	22 (0.28)	37 (0.48)	22 (0.28)	24 (0.31)	21 (0.28)	34 (0.43)
	v-v	9 (0.12)	12 (0.15)	1 (0.01)	4 0.05)	8 (0.10)	17 (0.22)
P-values	Allelic	0.6	0.71	0.41	0.65	0.75	0.59
	Genotypic	0.19 [0.32]	0.87 [0.70]	0.58 [0.76]	0.68 [0.66]	0.27 [0.45]	0.68 [0.71]

**Italy sCJD**							
Major allele count (frequences)		297 (0.76)	248 (0.64)	319 (0.82)	301 (0.78)	300 (0.77)	205 (0.53)
	wt	111 (0.58)	84 (0.44)	133 (0.70)	122 (0.64)	116 (0.62)	54 (0.28)
Genotype distribution (frequences)	wt-v	75 (0.39)	80 (0.42)	51 (0.28)	57 (0.30)	68 (0.36)	97 (0.51)
	v-v	6 (0.03)	28 (0.14)	5 (0.02)	12 (0.06)	4 (0.02)	40 (0.21)
P-values	Allelic	0.76	0.54	0.55	0.37	0.34	0.95
	Genotypic	0.25 [0.43]	0.75 [0.73]	0.80 [0.20]	0.60 [0.77]	0.14 [0.54]	0.87 [0.78]

**UK sCJD**							
Major allele count (frequences)		69 (0.72)	65 (0.68)	82 (0.85)	71 (0.74)	69 (0.72)	59 (0.61)
Genotype distribution (frequences)	wt	25 (0.52)	22 (0.46)	36 (0.75)	28 (0.58)	25 (0.52)	20 (0.42)
	wt-v	19 (0.40)	21 0.44)	10 (0.21)	15 (0.31)	19 (0.40)	19 (0.39)
	v-v	4 (0.08)	5 (0.10)	2 (0.04)	5 (0.11)	4 (0.08)	9 (0.19)
P-values	Allelic	0.37	0.36	0.47	0.13	0.3	0.19
	Genotypic	0.65 [0.42]	0.63 [0.47]	0.44 [0.17]	0.31 [0.62]	0.54 [0.31]	0.25 [0.15]

**Overall sCJD**							
Major allele count (frequences)		482 (0.76)	408 (0.64)	535 (0.84)	496 (0.78)	484 (0.78)	352 (0.56)
	wt	183 (0.58)	135 (0.43)	225 (0.71)	200 (0.63)	188 (0.60)	101 (0.32)
Genotype distribution (frequences)	wt-v	116 (0.36)	138 (0.43)	85 (0.27)	96 (0.30)	108 (0.35)	150 (0.47)
	v-v	19 (0.06)	45 (0.14)	8 (0.02)	21 (0.07)	16 (0.05)	66 (0.21)
P-values	Allelic	0.84	0.6	0.29	0.2	0.89	0.61
	Genotypic	0.92 [0.73]	0.85 [0.66]	0.57 [0.36]	0.46 [0.58]	0.90 [0.87]	0.84 [0.77]

**UK vCJD**							
Major allele count (frequences)		67 (0.66)	64 (0.63)	90 (0.87)	80 (0.78)	67 (0.67)	61 (0.60)
	wt	21 (0.41)	19 (0.37)	39 (0.75)	31 (0.61)	23 (0.46)	19 (0.37)
Genotype distribution (frequences)	wt-v	25 (0.49)	26 (0.51)	12 (0.23)	18 (0.35)	21 (0.42)	23 (0.45)
	v-v	5 (0.10)	6 (0.12)	1 (0.02)	2 (0.04)	6 (0.12)	9 (0.18)
P-values	Allelic	0.36	0.55	0.84	0.51	0.54	0.88
	Genotypic	0.28 [0.26]	0.39 [0.79]	0.99 [0.96]	0.80 [0.60]	0.55 [0.27]	0.65 [0.99]

P-values are not corrected for multiple testing

In brackets p-values adjusted by PRNP M129V genotype, age at onset and gender

wt: wild type

v: variant

sCJD: sporadic Creutzfeldt Jakob disease

vCJD:variant Creutzfeldt Jakob disease

The htSNPs genotyped allowed us to define the two major clades, H1 and H2, which have been described in Caucasians in several previous studies [32]. In order to facilitate comparisons we have adopted the same terminology for H1 sub-haplotypes as that used by Pittman *et al.*[22]. We did not find any evidence of association between any of the *MAPT *haplotypes and sCJD. We specifically did not find association with the subhaplotype H1c, which has been previously reported to be related to several neurodegenerative diseases. We also compared *MAPT *haplotypes between sCJD and vCJD from the UK. We found that the frequency of two rare haplotypes, H1f and H1j, were significantly different in sCJD (p = 0.04) and vCJD patients (p = 0.01) (Table 4). However, these two variants were present in a small proportion of patients, and after adjusting by the number of test performed (14 pairwise haplotypic comparisons were made) the results were not statistically significant (p = 0.56 for H1f) and (p = 0.14 for H1j). When we compared the frequency of these haplotypes in vCJD versus the Dutch control population, only the difference in H1j (3% in controls versus 7% in vCJD) was of borderline significance (p = 0.06).

**Table 4:** Haplotype analysis of the association between *MAPT *and Creutzfeldt-Jakob disease

**Haplotype**	**Alleles**						**Frequency**		***P*-value**	**Frequency**		***P*-value**
					
	**rs242559**	**rs242557**	**rs3785883**	**rs2471738**	**H1/H2**	**rs7521**	**Controls**	**sCJD**		**UK sCJD**	**UK vCJD**	
H1e	A	G	G	C	G	A	0.25	0.26	ref	0.24	0.2	0.51
H2a	C	G	G	C	A	G	0.23	0.22	0.64	0.28	0.31	ref
H1c	A	A	G	T	G	G	0.12	0.15	0.46	0.16	0.1	0.4
H1d	A	A	G	C	G	A	0.13	0.1	0.08	0.07	0.16	0.18
H1f	A	G	A	C	G	G	0.05	0.05	0.88	0.08	0.02	0.04
H1g	A	A	G	C	G	G	0.05	0.05	0.91	_	_	_
H1h	A	G	A	C	G	A	0.04	0.04	0.64	_	_	_
H1i	A	A	A	C	G	A	0.03	0.03	0.68	_	_	_
H1j	A	A	A	T	G	G	0.03	0.02	0.45	0.01	0.07	0.01
H1b	A	G	G	C	G	G	0.02	0.02	0.55	_	_	_

P-values are not corrected for multiple testing

Included all haplotypes present in both sCJD and vCJD

sCJD: sporadic Creutzfeldt Jakob disease

vCJD:variant Creutzfeldt Jakob disease

## Discussion

Our study assesses the relationship between *MAPT *haplotypic variations and CJD for the first time. To the best of our knowledge, there has only been one published article studying the role of *MAPT *in sCJD aetiology [33]. Sánchez-Valle et al. found no association between a polymorphism in exon 1 of *MAPT *in a group of 29 sCJD cases and 29 controls. We examined 6 htSNPs, which are part of an extended *MAPT *haplotype, in a Dutch population-based sample of sCJD patients and a cognitively normal control group of similar age distribution. Statistical analysis revealed no significant differences between cases and controls, with all p-values higher than 0.05 before correcting for multiple testing. Haplotype analysis still failed to detect any significant difference. We genotyped the same polymorphisms in two other sample groups of sCJD cases from Italy and the UK. All three populations of cases showed similar demographical characteristics. Due to selection bias in the low number of UK sCJD cases tested (n = 48), valine carriers were overrepresented in this subgroup. The frequencies of codon 129 genotypes in UK sCJD cases are similar to those found in other European countries. Our data do not show association between *PRNP *codon 129 and any of the *MAPT *SNPs. Therefore our conclusion of no association between *MAPT *and sCJD is not affected by the differences in *PRNP *genotypic distribution in the sample sets.

In our single locus analysis we did not find any significant difference between UK sCJD and vCJD patients. We found some *MAPT *haplotype frequencies unequally distributed, but none of the differences remained statistically significant after applying a multiple testing correction.

One possible caveat of our study is the fact that we included a proportion of clinically diagnosed cases (26% vCJD and 36% sCJD). However, it has been shown that "probable" cases fulfilling the clinical diagnostic criteria have a very high positive predictive value (98.5%) [34].

Lack of statistical power could be another explanation for our results. We think that this is unlikely. As a proof of principle, the association of sCJD with *PRNP *M129V genotypes in the overall sample yielded a very low p-value (<10^-11^). The sCJD risk of *PRNP *129 M homozygotes versus *PRNP *129 heterozygotes was 3.8 folds higher (95%CI from 2.6 to 5.5). Without multiple testing corrections we estimated that the statistical power of our genotypic analysis would be of 85% to detect an odds ratio as low as 1.6. In order to increase the power of the study, we pooled all sCJD cases. Therefore, although they were all Caucasians, in the second stage of the analysis not all sCJD cases and controls came from the same country. Another caveat of the study is the fact that the comparison between UK sporadic versus variant CJD was not age-matched. However, to the best of our knowledge, there are not *MAPT *age-related variations and, therefore, this should not be a major issue for our analysis.

## Conclusion

Our study shows no evidence for an association between *MAPT *gene variations and sCJD, and some weak evidence for an association to vCJD.

## Competing interests

The author(s) declare that they have no competing interests.

## Authors' contributions

PSJ performed the statistical analyses and drafted the manuscript. MB, AG & RK provided patient's samples and some genotypes from the UK, and reviewed critically the manuscript. GC & AP provided patient's samples and some genotypes from Italy, and reviewed critically the manuscript. AA performed most laboratory analyses. CvD reviewed critically the manuscript and contributed to its final version. All authors read and approved the final manuscript.

## Pre-publication history

The pre-publication history for this paper can be accessed here:


